# Mouse Model of Graves’ Orbitopathy in Current Research and Future Perspectives

**DOI:** 10.3390/medicina62050961

**Published:** 2026-05-14

**Authors:** Gyeong Min Lee, Wook Hyun Jung, Yeseul Kim, Myung In Oh, Sun Young Jang

**Affiliations:** 1 Department of Ophthalmology, Hallym University Kangnam Sacred Heart Hospital, Hallym University College of Medicine, 1, Singil-ro, Yeongdeungpo-gu, Seoul 07441, Republic of Korea; arips88@gmail.com; 2 Department of Ophthalmology, Soonchunhyang University Bucheon Hospital, Soonchunhyang University College of Medicine, 170, Jomaru-ro, Bucheon 14584, Republic of Korea; slayernada77@naver.com; 3 Department of Ophthalmology, Yonsei Eagle Eye Center, 39, Daewangpangyo-ro 606beon-gil, Bundang-gu, Seongnam 13524, Republic of Korea; rjqnrdl2060@hanmail.net; 4 Department of Medicine, Soonchunhyang University College of Medicine, 22, Soonchunhyang-ro, Sinchang-myeon, Asan 31538, Republic of Korea; om2624@naver.com

**Keywords:** Graves’ orbitopathy, Graves’ disease, disease models, animal, mice

## Abstract

Graves’ orbitopathy (GO) is a representative extrathyroidal manifestation of autoimmune thyroid disease. Despite extensive research, the precise pathogenic mechanisms remain incompletely understood. Moreover, the lack of an ideal animal model has limited in vivo investigation, with most studies relying on in vitro systems using orbital fibroblasts. In this review, we discuss currently available mouse models of GO and recent advances in experimental modeling strategies, aiming to provide an integrated framework for future research.

## 1. Introduction

Graves’ orbitopathy (GO) is a complex autoimmune disease characterized by inflammation, adipogenesis, and fibrotic remodeling of orbital tissue [1,2,3]. While understanding of the immunological mechanisms has advanced [4], effective treatments available in clinical practice remain limited. Systemic steroid therapy, currently the standard treatment for active GO, has variable efficacy, frequent side effects, and limited long-term prognosis, highlighting the critical need for novel therapeutic strategies [5,6].

The recent emergence of targeted immunomodulatory therapies is shifting the treatment paradigm for GO [7,8]. Mechanism-based drugs targeting specific pathways, such as B cell activation, cytokine signaling, and receptor-mediated immune responses, are showing promise, highlighting the importance of sophisticated preclinical models to support these therapies [4,7,8,9,10].

However, the development of an animal model for GO remains challenging. While hyperthyroidism, a systemic disease, can be induced with relative certainty, GO requires specific interaction between immune cells and orbital fibroblasts [11,12]. As a result, existing models only reproduce some characteristics, such as autoimmune levels or specific inflammatory responses, and are limited in that they fail to fully capture the complex clinical manifestations and pathological changes observed in actual patients [13,14].

These limitations are not due to the deficiencies of specific models, but rather reflect the pathological heterogeneity of GO itself [15]. Ultimately, finding a single model that perfectly recapitulates human GO is challenging [16]. Therefore, strategically selecting the optimal model is crucial, depending on whether the research goal is to elucidate mechanisms or validate therapeutic approaches.

Therefore, this review comprehensively analyzes existing GO mouse models, categorizing them by major induction strategies. Furthermore, we analyze the pathological phenotypes observed in each method and identify the gap between these experimental results and clinical practice, thereby providing a strategic framework for future research.

## 2. Literature Search Strategy

This narrative review was based on a literature search of PubMed and Google Scholar for studies published up to March 2026. Search terms included combinations of disease-related keywords such as “Graves’ orbitopathy,” “Graves’ ophthalmopathy,” and “thyroid eye disease,” together with model-related terms including “mouse model,” “animal model,” and “murine.” Additional keyword searches included “TSHR,” “IGF-1R,” “adenovirus,” “electroporation,” “Cre-loxP,” and related terms. Original studies and relevant review articles published in English were considered, with emphasis on experimental GO models and translational relevance.

## 3. Pathology of GO

GO is a tissue-specific autoimmune disorder characterized by pathological processes localized to the orbital connective tissue that are not solely explained by systemic thyroid status. Although GO is clinically associated with Graves’ disease, its orbital manifestations are driven by distinct local immune–stromal interactions [1,2]. Consequently, these site-specific mechanisms must be prioritized when evaluating animal models to ensure they accurately reflect the unique microenvironment of the human orbit.

A key feature of GO pathogenesis is the activation of orbital fibroblasts. They are a heterogeneous cell population that functions as both immunological targets and effector cells. Orbital fibroblasts express functional thyroid-stimulating hormone receptor (TSHR) and insulin-like growth factor-1 receptor (IGF-1R), which form a signaling complex that amplifies downstream inflammatory and metabolic pathways. Rather than acting independently, TSHR–IGF-1R cross-talk enhances hyaluronic acid synthesis, cytokine production, and metabolic remodeling, providing a mechanistic basis for the therapeutic efficacy of IGF-1R blockade [3,4]. When stimulatory autoantibodies bind to the TSHR, this receptor initiates a cascade of reactions that lead to excessive hyaluronic acid synthesis and interstitial edema. This process ultimately drives the pathological expansion of orbital connective tissues seen in GO patients [4,15].

Complementing these fibroblast-induced changes, the infiltration of activated T cells, B cells, macrophages, and fibrocytes critically modulates the orbital environment. These cells induce a dynamic cytokine cascade characterized in active disease by a Th1-dominant response associated with tumor necrosis factor-α and interferon-γ signaling, leading to acute inflammation and edema. As the disease progresses, increased Th2-associated and profibrotic signaling promotes adipogenesis and fibrotic remodeling [15,17]. Furthermore, B cells play a dual role in disease progression, facilitating both autoantibody-mediated responses and cellular immunity through antigen presentation and cytokine-mediated signaling [4,15,18].

Importantly, the pathological landscape of GO is highly variable, with the extent of inflammation, adipogenesis, and fibrosis differing markedly among patients [2,15]. This pathological heterogeneity indicates that GO does not follow a single linear pathogenic pathway, but rather reflects multiple overlapping and partially independent disease processes.

From an animal modeling perspective, these characteristics suggest that reproducing individual components, such as hyperthyroidism or autoantibody production, does not necessarily equate to accurate modeling of GO. Because the utility of a model depends on the specific orbital changes it represents, researchers must strategically tailor the model’s pathologic profile to their specific research goals to achieve meaningful experimental results.

## 4. Challenges in Modeling GO and Current Experimental Approaches

**Figure 1:**
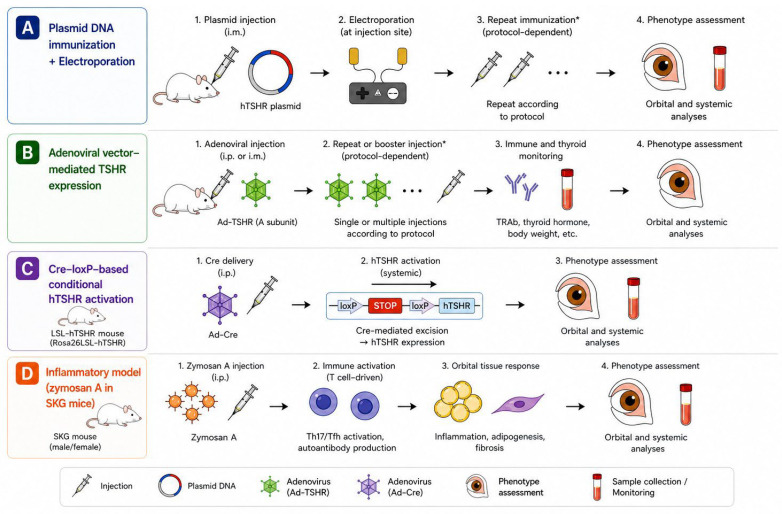
Representative induction workflows of major GO mouse models. Schematic overview of major induction strategies used in GO mouse models: (**A**) plasmid DNA immunization with electroporation, (**B**) adenoviral vector-mediated TSHR expression, (**C**) Cre–loxP-based conditional hTSHR activation, and (**D**) inflammatory induction using zymosan A in SKG mice. Final assessments may include orbital and systemic phenotyping. The timing and number of repeated immunizations or injections vary among studies. GO, Graves’ orbitopathy; TSHR, thyroid-stimulating hormone receptor; hTSHR, human TSHR; i.m., intramuscular; i.p., intraperitoneal. * Protocol-dependent step; injection frequency and interval vary among studies.

To critically assess the translational relevance of experimental models, it is essential to first define the key phenotypic endpoints that characterize human GO [2,3,4]. Given that GO is a complex, heterogeneous disorder involving overlapping inflammatory, metabolic, and structural shifts, no single parameter can fully capture its clinical reality [15,16]. Primary endpoints should include reproducible evidence of disease induction, such as thyroid hormone abnormalities, TSHR autoantibody generation, inflammatory cell infiltration, adipogenesis, and hyaluronic acid accumulation within orbital tissues [18,19]. Secondary endpoints should reflect more advanced and clinically relevant structural manifestations, including extraocular muscle enlargement, chronic fibrosis, and objectively measurable proptosis [19]. These higher-order phenotypes remain more difficult to reproduce consistently in current mouse models, yet are particularly important for translational relevance. These considerations provide a practical framework for interpreting the strengths and limitations of currently available GO mouse models. Representative induction workflows of the major GO mouse models are summarized in Figure 1.

### 4.1. The Gap Between Thyroid Dysfunction and Orbital Disease

GO presents with clinical differences between the systemic and ocular manifestations of Graves’ disease. This makes it difficult to fully recapitulate the disease phenotype in an experimental model [16]. Indeed, hyperthyroidism is elicited with relatively high efficacy, whereas the concomitant ophthalmopathy tends to be less prevalent and less reproducible [11,13]. These differences suggest that GO is not simply a secondary consequence of thyroid dysfunction, but rather a tissue-specific autoimmune disease driven by immune mechanisms unique to the orbital microenvironment [15].

GO modeling is challenging due to the chronic course of the disease and the diverse phenotypes that vary from patient to patient. In humans, inflammation, adipogenesis, and fibrosis occur at different times, and their severity also varies significantly from person to person [2]. Because of these characteristics, it is virtually impossible to reproduce the entire disease process within a single model. Most short-term experiments tend to focus on the early inflammatory phase, often failing to demonstrate late-stage symptoms, such as adipose tissue proliferation, fibrosis, and extraocular muscle hypertrophy, seen in real patients. Therefore, the underrepresentation of ophthalmopathy in experiments should be understood as stemming from the complex nature of the disease itself, rather than a problem with modeling techniques. Consequently, this supports the need for a strategic approach targeting specific pathological stages, depending on the research objective, rather than replicating the entire disease [14,16].

### 4.2. Plasmid-Based Genetic Immunization

To address the low prevalence of orbital symptoms in experimental models, early studies employed genetic immunization strategies targeting the TSHR to induce autoimmune responses [11,13]. Plasmid-based immunization using full-length TSHR or the immunodominant A subunit reliably induces TSHR autoantibodies and hyperthyroidism [19,20]. This is particularly effective when combined with in vivo electroporation to enhance antigen expression and immune activation [21]. Recent advancements have optimized this process by administering cardiotoxin five days prior to the first vaccination to enhance myofiber regeneration and plasmid uptake. The recombinant plasmid is injected into the target muscle—such as the biceps femoris—followed by immediate electrical stimulation with an electroporator. This vaccination is typically repeated three times at 4-week intervals. Successful immunization in this model is characterized by significant elevations in serum T3, T4, and TRAb levels, accompanied by clinical proptosis in approximately 50% of the subjects. Histopathological examination further reveals hallmark GO features, including retrobulbar inflammation, adipogenesis, and increased expression of inflammatory markers such as interleukin-1β (IL-1β) and interleukin-6 (IL-6) [22]. However, despite this potent systemic autoimmunity, orbital phenotypes remain inconsistent. Only a subset of animals displays retrobulbar inflammation or early adipogenic shifts, whereas the hallmark fibrotic remodeling is rarely achieved [11,21].

### 4.3. Viral Vector-Based Delivery Systems

To overcome the inherent limitations of plasmid-based immunization—namely, the low and inconsistent incidence of orbital phenotypes—researchers subsequently turned to viral vector-based delivery systems [11]. Among these, adenoviral vectors expressing the TSHR A-subunit (Ad-TSHR) emerged as a potent strategy to amplify immune responses [20,21]. Adenoviral delivery enables robust antigen expression in vivo and induces a stronger and more sustained immune cascade compared with naked DNA plasmids [21,23].

Recent refinements, such as repeated Ad-TSHR injections, have yielded a higher incidence of orbital involvement, including significant adipogenesis and early fibrotic changes. However, despite enhanced immune stimulation, these models still do not fully recapitulate the entire human GO spectrum. Significant variability across different laboratories and the practical burden of repetitive dosing remain key challenges [16,23]. For instance, some protocols require up to nine injections of Ad-TSHR to achieve clinical features of GO in 70% of models, posing a significant practical burden and requiring longer induction periods than plasmid-based methods [24]. Ultimately, these limitations underscore that simply amplifying systemic autoimmunity is insufficient to reliably mirror the site-specific pathological complexities of the disease [14,25,26].

### 4.4. Cell-Based Immunization Strategies

Wang et al. [27] compared two animal models of GO: recombinant adenovirus expressing the human thyrotropin receptor A subunit (Ad-TSHR A) gene immunization and dendritic cell (DC)-based immunization. In their study, DCs were cultured and subsequently infected with a recombinant adenovirus encoding TSHR and interferon-γ (IFN-γ). Cellular immunization was performed in mice at 3-week intervals. As professional antigen-presenting cells, DCs play a pivotal role in initiating adaptive immune responses. Notably, the modeling rate achieved with DC-based immunization was approximately 60%, suggesting that although immune cell priming enhances GO induction, it may still be insufficient to fully reproduce the complete spectrum of the disease phenotype.

In parallel, T cell-driven models further highlight the critical role of cellular immunity in GO pathogenesis [28]. Park et al. [29] introduced a zymosan A-treated SKG mouse model, in which SKG mice were intraperitoneally injected with zymosan A at 8 weeks of age. Notably, these mice exhibited enhanced orbital adipogenesis, along with GO-like inflammatory adipose tissue phenotypes induced by T cell-mediated autoimmune responses.

### 4.5. Conditional Gene Targeting Using the Cre–loxP System

The paradigm for modeling GO has evolved from a focus on immune intensity to mechanistic accuracy [14,16]. Previous attempts to induce orbital symptoms by amplifying the systemic immune response through immune-based models have yielded inconsistent and limited results [11,13]. This discrepancy underscores that GO is not a disease that can be faithfully reproduced by sheer immune strength alone [2,15,25]. Instead, it is a complex condition that can only be understood when the specific biological conditions necessary for its onset are precisely met [15]. This shift in perspective has catalyzed the transition toward conditional gene targeting via the Cre–loxP system [14,16].

Unlike plasmid- or viral vector-based strategies that prioritize the magnitude of systemic immune responses, the Cre–loxP system facilitates conditional gene targeting through a more refined approach [30]. It allows predefined genetic sequences to be selectively activated or deleted within specific cell populations and at distinct time points. This approach permits a precise dissection of how localized and temporally restricted signaling events drive disease pathogenesis, shifting the modeling paradigm from broad immune stimulation toward the targeted interrogation of cell-specific immune–stromal interactions [26]. In the context of GO, such precision-oriented control provides a robust conceptual framework for isolating the distinct contributions of orbital fibroblast lineages to inflammation, adipogenesis, and tissue remodeling, all while minimizing the confounding variables associated with systemic immune overactivation [14,16,25].

A recent study by Bao et al. (2023) [30] exemplifies how this conceptual precision can be implemented in practice. By integrating the Cre–loxP system with targeted genomic insertion at the Rosa26 locus, the authors established a conditional GO mouse model in which a single administration of a Cre-expressing adenovirus was sufficient to induce both systemic thyroid autoimmunity and orbital pathological features. This streamlined approach demonstrates that precise genetic triggers can effectively elicit GO manifestations, highlighting the utility of conditional control in disease modeling [30]. However, despite this important advance, the model remains constrained by limited orbital specificity, incomplete representation of fibroblast heterogeneity, and insufficient recapitulation of chronic disease progression and extraocular muscle involvement [14,16,25].

The diverse experimental strategies discussed above, ranging from traditional genetic immunization to recent genetic engineering approaches, are summarized in Table 1. To enable practical cross-study comparison, the included mouse models were qualitatively assessed according to systemic (1st endpoint) and orbital (2nd endpoint) phenotype penetrance. In addition, key advantages, major limitations, and the most suitable research applications of each model are outlined to guide model selection according to specific experimental objectives. This comparative framework underscores the distinct strengths and translational relevance of currently available GO models.

**Table 1:** Comparison of experimental approaches for induction of Graves’ orbitopathy.

Reference	Model Strategy	Mouse Strain (Sex)	Method	Duration	1st Endpoints	2nd Endpoints	Phenotype Penetrance (1st/2nd)	Key Advantages	Limitations	Best Use
Moshkelgosha et al. (2013) [21]	Genetic immunization (Plasmid)	BALB/c (F)	TSHR A-subunit plasmid + EP	12 wk (4× q3w)	TSHR Ab induction with hypothyroid-predominant response	Inflammation, adipogenesis, EOM enlargement, proptosis, fibrosis	Partial/High	Robust multi-domain orbital phenotype with MRI validation	Predominantly hypothyroid response and labor-intensive induction	Studies of orbital remodeling and inflammation-to-fibrosis progression
Xia et al. (2017) [19]	Genetic immunization (Plasmid)	BALB/c (F)	hTSHR A-subunit plasmid + EP	12 wk (4× q3w)	Hyperthyroidism, TRAb elevation	EOM enlargement, HA accumulation, mild inflammation	High/Partial (>80% thyroid phenotype)	Reproducible hyperthyroidism with measurable orbital involvement	Limited proptosis and modest inflammatory phenotype	Studies of systemic thyroid autoimmunity with early orbital changes
Nagayama et al. (2002) [20]	Viral vector (Adenovirus)	BALB/c (F/M), multiple strains (mainly F)	Intramuscular Ad-TSHR injection	9–14 wk (3× q3w)	Hyperthyroidism, T4 elevation, TSI/TBII, goiter	Minimal orbital assessment	High/Limited (25–55%)	Strong thyroid autoimmunity induction across strains	Poorly defined orbital phenotype	Studies of thyroid autoimmunity, TSHR antibody biology, and host susceptibility
Wang et al. (2023) [27]	Cellular/Viral immunization	BALB/c (F)	TSHR + IFN-γ–modified dendritic cells (DC)/repeated Ad-TSHR A-subunit injections	22 wk (DC)/41 wk (Ad-TSHR)	Hyperthyroidism, free T4 elevation, TRAb induction, thyroid hyperplasia	Proptosis, EOM hypertrophy, adipogenesis, HA accumulation, fibrosis	Partial/Partial (DC, 60%); High/High (Ad, 72%)	Direct comparison of two high-yield GO induction strategies	Long induction period and protocol complexity	Studies of comparative model selection and immune pathway-specific GO mechanisms
Bao et al. (2023) [30]	Genetic engineering	BALB/c-backcrossed (M/F)	Cre-loxP-based hTSHR activation + TAT-Cre injection	14 wk (single induction)	Hyperthyroidism, T4/fT4 elevation, TSHR Ab/TSAb induction, goiter	EOM enlargement, retrobulbar adipogenesis, macrophage infiltration, fibrosis	High/High (81.25%)	Single-induction GD/GO model with robust systemic and orbital phenotypes	Requires transgenic platform; limited proptosis and HA phenotype	Studies of rapid GD/GO induction, disease mechanisms, and therapeutic screening
Park et al. (2020) [29]	T cell-mediated autoimmune model	SKG/BALB/c controls	Single zymosan A intraperitoneal injection	12 wk (single dose)	No thyroid phenotype assessed	Proptosis, orbital adipogenesis, inflammatory infiltration, blepharitis	Limited/Partial	Robust T cell-mediated orbital inflammatory phenotype	Non-TSHR mediated; no hyperthyroidism	Studies of immune-driven orbital inflammation and adipogenesis
Wu et al. (2024) [23]	Dual-antigen immunization (Plasmid)	BALB/c (F)	TSHR A-subunit + IGF-1Rα plasmids with EP	32–43 wk (12 injections)	Hyperthyroidism, T4 elevation, TSAb/TSBAb induction, goiter	Proptosis, EOM enlargement, adipogenesis, inflammatory infiltration, HA accumulation	High/High (83.3%)	Incorporates TSHR–IGF-1R crosstalk with robust dual phenotype	Prolonged repeated immunization protocol; small group size	Studies of receptor crosstalk, advanced GD/GO induction, and therapeutic testing
Moshkelgosha et al. (2021) [31]	Microbiome-modulated plasmid immunization model	BALB/c (F)	Gut microbiota modulation + TSHR-A plasmid immunization	22 wk	TSAb/TRAb induction, T4 elevation, thyroid hyperplasia	Orbital BAT expansion, extraocular muscle atrophy, clinical proptosis/inflammation	High/High (84%, 70%)	Demonstrates microbiome-dependent modulation of GD/GO phenotype	Complex multi-arm design; limited fibrosis and inflammatory phenotype	Studies of host–microbiome interactions and environmental modifiers in GO
Kim et al. (2024) [22]	Drug-testing application of plasmid GO model	BALB/c (F)	TSHR plasmid immunization + EP followed by oral ibrutinib	14 wk total (2-wk treatment phase)	T3, T4 elevation, TRAb	Proptosis, orbital inflammation, adipogenesis, EOM inflammatory change	High/Partial (50% proptosis)	Validates therapeutic responsiveness in an established GO model	Primarily treatment study; limited MRI/quantitative orbital structural assessment	Studies of preclinical drug testing and inflammatory pathway targeting

TSHR, thyroid-stimulating hormone receptor; IGF-1R, insulin-like growth factor-1 receptor; TRAb, thyrotropin receptor antibody; TSAb, thyroid-stimulating antibody; TSBAb, thyroid-stimulation blocking antibody; TSI, thyroid-stimulating immunoglobulin; TBII, thyrotropin-binding inhibitory immunoglobulin; T3, triiodothyronine; T4, thyroxine; fT4, free thyroxine; F, female; M, male; GO, Graves’ orbitopathy; GD, Graves’ disease; EOM, extraocular muscle; HA, hyaluronic acid; BAT, brown adipose tissue; EP, electroporation; CTX, cardiotoxin; DC, dendritic cell; Ad, adenovirus. Phenotype penetrance (1st/2nd) indicates qualitative penetrance of systemic (1st) and orbital (2nd) disease features, respectively. Grades were defined as High (commonly observed multidomain phenotypes), Partial (reproducible but limited features), Variable (heterogeneous findings), or Limited (minimal involvement).

## 5. Conclusion and Future Perspectives

### 5.1. Current Limitations: The Gap Between Models and Clinical Reality

Developing an animal model that faithfully reflects the clinical reality of GO remains challenging. A key issue is the persistent gap between experimental findings and the complex, long-term disease course observed in patients. While many existing models successfully capture early inflammatory changes, they often fail to reproduce later-stage features such as chronic fibrosis and extraocular muscle enlargement, both of which are closely associated with clinical outcomes.

Several factors likely account for these limitations. First, inherent species differences between mice and humans cannot be overlooked. These include not only anatomical discrepancies but also differences in immune responses, particularly in orbital fat composition and TSHR expression within the orbital microenvironment. Second, reproducibility remains a concern. Across studies, the incidence and severity of orbital manifestations—such as proptosis—vary considerably, making it difficult to directly compare results or establish standardized protocols. Third, current models tend to simplify the disease process. Many rely on a single immunological trigger, most commonly TSHR immunization, which does not fully reflect the multifactorial nature of GO. Environmental influences, including smoking, oxidative stress, and alterations in the microbiome, are rarely incorporated despite their known clinical relevance. Finally, incomplete phenotypic representation remains a recurring issue. Some models, particularly viral or plasmid-based systems, efficiently induce hyperthyroidism but show limited or inconsistently evaluated orbital involvement, falling short of reproducing the tissue-specific pathology observed in patients.

Moving forward, improving the translational value of GO models will likely require a more integrated approach. Rather than relying on a single initiating factor, future models may need to incorporate both genetic susceptibility and clinically relevant environmental modifiers to better reflect disease complexity.

### 5.2. Integration of Genetic Precision and Environmental Factors

The introduction of conditional gene targeting technologies, such as the Cre-loxP system, has significantly improved the accuracy of GO models [30]. However, the actual clinical manifestations of GO are not determined solely by genetic predisposition. Genetic susceptibility and external environmental factors interact in a complex manner to trigger disease onset. Therefore, future modeling studies should adopt a “multi-hit” framework that combines precise gene regulation with various immune modulators.

In this context, specific inflammatory models offer crucial complementary insights. A prime example is the Zymosan A-induced model [29], which recapitulates T cell-driven orbital inflammation and adipogenesis despite the absence of systemic TSHR antibody-mediated autoimmunity. The fact that key features of orbital symptoms can be recapitulated without the presence of TSHR antibodies suggests that local immune dysregulation within the orbital microenvironment is a powerful and independent factor in determining GO pathophysiology. This underscores the need to move beyond the single marker of systemic antibody levels to focus on the complex intercellular interactions occurring within the orbital stroma.

Furthermore, a recent study with Ibrutinib [22] demonstrated that targeting specific signaling pathways (e.g., BTK/ITK) within this multifactorial model can significantly suppress orbital inflammation [22]. This demonstrates that combining local inflammatory stimuli or environmental stressors with precise gene regulation models can more clearly identify therapeutic targets applicable to clinical practice.

Future studies will need to combine the precision of the Rosa26-targeted Cre-loxP system with the sensitivity of local inflammatory stimuli or environmental stressors. This multi-hit framework will allow for a more faithful recapitulation of the spectrum of GO, focusing on the complex, tissue-specific nature of the disease, rather than simply inducing hyperthyroidism.

Ultimately, next-generation GO models should not only recapitulate disease characteristics but also serve as platforms for identifying symptom-specific therapeutic targets. Precision genetic approaches that selectively model adipogenesis or fibrosis pathways could help discover therapeutic strategies tailored to the diverse clinical phenotypes of GO, thereby bridging the gap between experimental results and clinical application.

### 5.3. Closing Remarks: Toward Personalized Precision Medicine in GO

**Figure 2:**
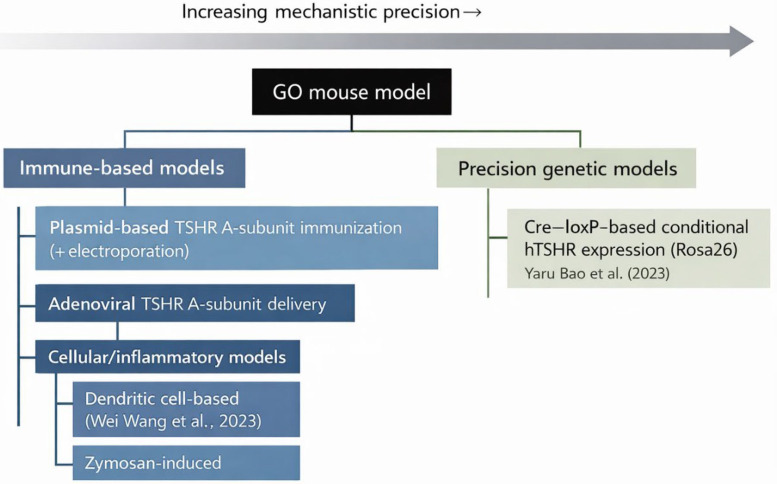
Evolution of experimental strategies for modeling Graves’ orbitopathy in mice. TSHR, thyroid-stimulating hormone receptor; hTSHR, human TSHR. (Wei Wang et al. [27]; Yaru Bao et al. [30]).

In summary, GO modeling has undergone a fundamental shift from simple systemic immune stimulation to a mechanism-centered, precise approach. As seen in the evolution of experimental strategies (Figure 2), the academic community is now focusing on the sophisticated reproduction of the microscopic biological environment required for the manifestation of orbital diseases, moving beyond merely enhancing immune responses.

The incorporation of advanced genetic tools, including the Rosa26-targeted Cre–loxP system, together with clinically relevant and stage-specific endpoints, represents a meaningful step toward improving the translational relevance of GO models. Although several challenges remain—such as the limited representation of chronic fibrosis and persistent species-specific differences—these next-generation approaches offer a more flexible platform for evaluating emerging therapies. This includes targeted strategies such as Bruton’s tyrosine kinase (BTK) inhibitors (e.g., ibrutinib), as well as potential anti-fibrotic interventions.

These refined models may also help bridge the gap between preclinical findings and clinical application. By better reflecting disease mechanisms observed in patients, they provide a more reliable framework for testing therapeutic hypotheses. Ultimately, continued refinement of these models may contribute to the development of more personalized treatment strategies, particularly for patients with severe or treatment-resistant GO.

## Data Availability

Data sharing is not applicable to this article, as no new data were created or analyzed in this review.

## Abbreviations

GO	Graves’ orbitopathy
TSHR	Thyroid-stimulating hormone receptor
IGF-1R	Insulin-like growth factor-1 receptor
IL	Interleukin
